# Prevalence of Infertility, Associated Factors, and Treatment Seeking Among Reproductive Age Couples in Merhabete Woreda, Northshewa Ethiopia, 2023: A Cross‐Sectional Study

**DOI:** 10.1002/hsr2.72224

**Published:** 2026-04-02

**Authors:** Hailegiyorgis Geleta Abocherugn, Abera Mamo Dibabu, Dereje Zeleke Belachew, Desalegn Girma, Amare Workie Gashu, Melese Wagaye Zergaw

**Affiliations:** ^1^ Department of Midwifery College of Medicine and Health Sciences Mizan Tepi University Mizan Teferi Ethiopia; ^2^ Departments of Midwifery College of Medicine and Health Sciences Wollo University Dessie Ethiopia; ^3^ Department of Nursing Asrat Woldeyes Health Science Campus Debre Berhan University Northshoa Ethiopia

**Keywords:** associated factors, Ethiopia, infertility, treatment seeking

## Abstract

**Background and Aims:**

Infertility is a significant public health problem that affects approximately 10%–15% of couples worldwide. Although it affects men and women equally, women often bear disproportionate social blame, which may result in reduced social status. In many settings, infertility is considered a socially acceptable reason for divorce in many settings. However, its prevalence varies considerably across countries and communities and is influenced by multiple demographic, behavioral, and medical factors. Despite its adverse consequences and the availability of medical treatments, community‐based evidence remains limited. Therefore, this study aimed to assess the prevalence of infertility, associated factors, and treatment‐seeking behavior among couples of reproductive age in Merhabete Woreda, North Shewa, Ethiopia, 2023.

**Methods:**

A community‐based cross‐sectional study was conducted from April 1, 2023, to May 30, 2023, among 846 couples in the Merhabete Woreda. Multistage sampling was performed, and data were collected using a semi‐structured interviewer‐administered questionnaire weight and height measuring scales. The data were entered into EpiData version 4.6. Data were analyzed using the Statistical Package for the Social Sciences, version 26. Bivariable and multivariable logistic regression were performed, and variables with a *p*‐value < 0.05 were considered significant. Model fit was evaluated using the Hosmer–Lemeshow test.

**Results:**

The prevalence of infertility was 23.4% (95% CI: 20.4–26.5), and 53.5% of infertile couples never sought medical help. Females aged 40–49 years (AOR: 7.3, 95% CI: 2.97–17.9), with no formal education (AOR: 3.92, 95% CI: 1.88–12.89), couples with a history of sexually transmitted infections (AOR: 3.82, 95% CI: 2.18–9.78), high stress (AOR: 3.97, 95% CI: 3.44–15.38), overweight (AOR: 2.73, 95% CI: 1.14–6.52), and obese (AOR: 3.12, 95% CI: 1.77–8.2) were significantly associated with infertility.

**Conclusions and Recommendations:**

The prevalence of infertility in this community exceeded global estimates, and more than half of the affected couples did not seek medical care. Integrating infertility screening into primary healthcare services, strengthening sexually transmitted infection (STI) prevention, promoting stress reduction and weight management, and enhancing community awareness may improve fertility outcomes and healthcare‐seeking behavior.

**Abbreviations**
AOR: adjusted odds ratio
ART: assisted reproductive technology
CI: confidence interval
COR: crude odds ratio
LIC: low income country
RAG: reproductive age groups
SSA: sub‐Saharan Africa
STIs: sexual transmitted infections
WHO: World Health Organization

## 1 Introduction

Infertility is a disorder of the male, female, or reproductive systems of both sexes and is defined as the inability to conceive after 12 months or more of regular, unprotected sexual intercourse [1]. Reproduction is highly valued among couples, and children are of great importance. However, infertility can be devastating and destructive to couples' dreams of having children. This is a major concern, particularly in the African context, including Ethiopia, as it negatively affects social relationships and marital institutions [2, 3]. Among those attempting conception, approximately 50% of women will become pregnant at 3 months, 75% at 6 months, and more than 85% by 1 year [4].

Infertility has several etiologic and contributory variables; however couples from developing nations, notably those in sub‐Saharan Africa, regard it as a punishment for some societal violations [5]. The etiologies include male‐related, ovulatory, tubal/uterine, other, and unexplained factors, accounting for 25%, 27%, 22%, 9%, and 17%, respectively [4]. Age, educational level, employment, alcohol intake, smoking, obesity, stress, and sexually transmitted infections (STIs) are major factors contributing to infertility [4, 5].

In Africa, approximately 40% of infertility cases are due to male factors, another 40% to female factors, 15% involve both partners, and the remaining 5% are unexplained [6]. The specific impact of some of these factors on infertility remains unclear, and further research is required in this regard [7]. Therefore, understanding the prevalence, associated factors, and treatment‐seeking behavior is important, as these are not well documented in Ethiopia, including in this study area.

Infertility is a major reproductive health problem, considered one of the world's most serious public health problems, and is regarded as an unsolved problem in the human race [8]. Globally, the prevalence of infertility has changed significantly in recent years, with estimates ranging from 10% to 15%, the trend of infertility varies widely across the globe, with between 48 million and 186 million people of reproductive age affected [1, 4]. Infertility affects approximately one‐tenth of the world's population with the lowest rates in Australia and highest in Africa [9]. The distribution of infertility cases differs between nations: 10% in the United States of America, 6% in the United Kingdom, and 25% in China [10]. The infertility belt, defined as a geographical area with a high prevalence of infertility, runs from West Africa to Central and East Africa [1]. Infertility in developing nations rises exponentially from 5% at 20–24 years of age to 62% between 45 and 49 years of age [11]. However, in sub‐Saharan Africa, the prevalence of infertility is as high as 30%.

Infertility has numerous consequences for individuals and couples in their communities. Childless couples are frequently disrespected, causing separation or divorce, ignorance, depression, anxiety, and low self‐esteem, ultimately resulting in suicide. In many cultures, failure to bear children is an acceptable reason for divorce [12]. Generally, infertility has severe social, psychological, and economic consequences for both men and women. In addition, socially stigmatized people are discouraged from positions of leadership, thereby wasting the opportunity to live again [13]. Concerns over life instability and being judged by others, violent behavior at home, which is associated with polygamous relationships, to rise the probability of having children [14].

Infertility rates often differ among populations and are influenced by social contrasts, degree of sexual behavior, the predominance of STIs, and reproductive activities [2]. Most couples perceive childlessness to be associated with evil spirits, ancestral curses, and promiscuity [6]. Factors related to infertility include maternal age at menarche [15], educational level, and alcohol consumption [16]. Obstetric history, genital infections [17], and body mass index (BMI) [18] have been identified as factors influencing fertility in high‐income countries (HIC).

The occurrence of STIs and, in some cases, other infections that occur after abortion or childbirth, or systemic infections may be important [11]. To overcome the problem of infertility, many couples engage in multiple sexual partners and polygamous relationships, which are linked to the risk of acquiring STIs [14]. Some try traditional or herbal remedies while waiting for supernatural power without any trial [8]. Infertility has serious social, psychological, and economic impacts on both men and women, and stigma can prevent affected individuals from assuming leadership roles, limiting their opportunities [4]. However, studies suggest that approximately half of infertile couples visit health facilities to seek infertility care [10].

The availability and accessibility of ART are also limited in developing nations, and the implementation of appropriate infertility treatments is currently not the main goal for most developing countries or international nonprofit organizations [14]. Infertility has serious social, psychological, and economic impacts, and stigma can limit affected individuals' participation in leadership and society [19].

Identifying infertility factors is key for prevention and care, yet it is largely neglected in many developing countries where reproductive health focuses on reducing birth rates [20]. Fortunately, it is possible to avoid a significant proportion of the risks associated with infertility in Africa if the prevalence and associated factors are well understood [1]. However, there are limited community‐based studies and a paucity of data on infertility factors in low‐income countries (LICs) [14].

Despite the growing social and public health significance of infertility in Ethiopia, evidence on its prevalence, associated factors, and treatment‐seeking behavior remains limited, particularly at the woreda level. Most existing studies are institution‐based, rely on secondary data, and focus primarily on women, overlooking the couples' involvement. Therefore, this community‐based study couples in Merhabete Woreda provides essential local evidence to inform prevention strategies, targeted interventions, and context‐specific health policy decisions.

## 2 Materials and Methods

### 2.1 Study Design and Setting

A community‐based cross‐sectional study was conducted from April 1, 2023, to May 30, 2023 in Merhabete Woreda, Northshewa, Ethiopia. It is one of the 28 woredas found within the Northshewa Zone in the Amhara regional state. Ensaro borders Merhabete to the south by the Oromia Region, to the north by Mida Woremo, to the east by Menz Keya Gebreal, and to the southeast by Moretna Jiru, with a total boundary of 1058.19 km^2^. The Jemma River defines the southern and eastern boundaries of the Woreda, whereas its tributary, the Wenchit River, defines its western and northern boundaries. The administrative center is Alem Ketema town, 183 km away from Addis Ababa, the capital city of Ethiopia. The woreda has a total population of 120,738, of whom 63,353 are men and 57,385 are women, with 66,664 (55.2%) aged between 15 and 49 years. The Merhabet woredas comprise 24 kebeles [21]. Woreda has one government hospital, six government and five private clinics, 23 health posts, five pharmacies, and a total of 54 health extension workers [22].

### 2.2 Study Participants and Eligibility Criteria

The source population included all couples of reproductive age couples living in Merhabete Woreda, Northshewa, and central Ethiopia, and all couples of reproductive age living for at least 1 year in eight selected kebeles of Merhabete Woreda, Northshewa, Ethiopia, during the study period were included in the study. Partners with permanent contraception (tubal ligation, vasectomy) and couples less than 12 months from the first post‐delivery or post‐abortion menses were excluded.

### 2.3 Sample Size Determination

The sample size was determined using a single population proportion for a finite population with an assumption of 95% confidence levels (*Z* = 1.96), 5% marginal error (*d* = 0.05), and a prevalence (p) of infertility of 50% and a 10% non‐response rate. The sample size was calculated as follows:

$$\mathrm{ni}=\frac{Z({\frac{\alpha }{2})}^{2}\times p\left(1-p\right)}{d^{2}}=\frac{({1.96)}^{2}\times 0.5\left(1-0.5\right)}{{\left(0.05\right)}^{2}}=384$$

A design effect of 2 was considered (2 × 384), resulting in a sample size of 768. Finally, after adding a 10% none response rate, the final sample size was 846.

### 2.4 Sampling Technique and Procedure

From 24 kebeles found in Merhabete woredas, eight kebeles, namely Harogenda, Fetra, Koso‐terehna, Kora‐wenchit, Afezez‐berkato, Geren, Buyo‐gedejewa, and Yesa‐ssamba, were selected using a simple random sampling (lottery) method. The required sample size was proportionally allocated to each group. Finally, the study unit was selected using a systematic random sampling method after determining the *K*th interval (*N*/*n* = *k*), which was 8. Where “*N*” is the total number of reproductive age group couples living in each selected kebeles, “*n*” is the sample size allocated proportionally for each kebele, and “*k*” is the skip interval.

The first study unit was selected using lottery methods out of 1 through *k* [8] and continued every eighth until we obtained the assigned sample size for each kebele.

**FIGURE 1:**
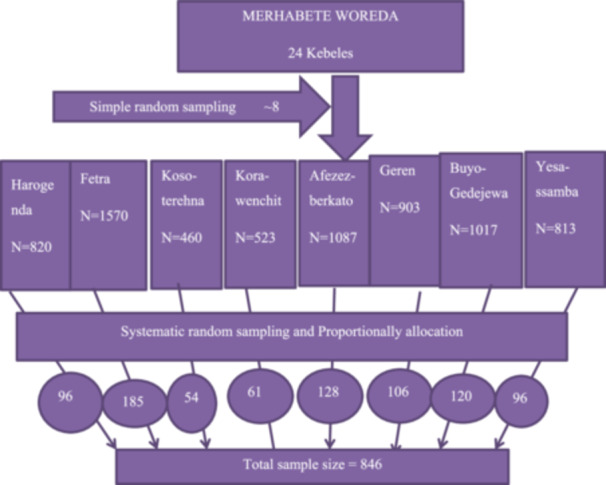
The schematic representation of the sampling procedure among reproductive age group couples living in Northshewa, Central Ethiopia, 2023.

The detailed sampling procedure is illustrated in Figure 1.

### 2.5 Operational and Term Definitions

Infertility: Infertility was defined as currently infertile if they did not become pregnant after exposure to the risk of conception during the previous 12 months [1, 10, 23, 24].

Risk of conception: was declared when all the following four conditions were fulfilled. (i) Want to have children. (ii) Did not use any form of birth control within the past 12 months. (iii) Reported having adequate sexual intercourse in the past 12 months and (iv) trying to become pregnant with their current partner for more than or equal to 1 year [10, 23]. Those who answered “YES” to all four questions were considered as infertile.

Treatment‐seeking: In this study, treatment‐seeking was defined as couples who visited health institutions for infertility treatment at least once [25].

Adequate coital frequency: was defined as couples with a coital frequency greater than or equal to 2 times per week [20].

Irregular menstrual cycle: when the menstrual cycle length was less than 21 days and/or more than 35 days [26].

Menstrual blood flow: classified as minimal menstrual flow (< 2 pads/day), moderate menstrual flow (2–4 pads/day), and heavy menstrual flow (> 4 pads/day) used during the menstrual period [26].

Substance use: an intentional ingestion of one or more psych stimulant drugs (alcohol, cigarette smoking, and chat) [27].

Tobacco use: In this study, couples who had smoked >=100 cigarettes in their lifetime and/or were currently smoked considered as current smokers, and couples who had never smoked, or who had smoked less than 100 cigarettes in their lifetime and were not currently smoking were considered noncurrent smokers [28].

Alcohol use: In this study, alcohol use was defined as past‐year abstainers when couples reported no lifetime drinking or less than five drinks per week of any amount, and heavy drinkers: when any alcohol consumed in any amount five or more drinks per week [29].

Coffee use: was defined as a user when couples consumed five or more cups per day, and non‐user when no or less than five cups per day were consumed [30].

Physical activity: In this study, vigorous‐intensity physical activities were defined as at least 150 min per week, such as heavy lifting, digging, aerobics, plaguing, or fast bicycling. Moderate‐intensity physical activity: Physical activity of at least 300 min per week, such as carrying light loads, bicycling at a regular pace, and walking. Sedentary physical activity: Less than the recommended physical activity of moderate‐intensity physical activity [31].

Stress: PSS scores of 0–13 were considered low stress, scores of 14–26 were considered moderate stress, and scores of 27–40 were considered high stress [32].

BMI: was calculated as weight in kilograms divided by height in square meters and interpreted as underweight (BMI < 18.5), normal (BMI 18.5–24.9), overweight (BMI 25.0–29.9), and obese (30.0) [33].

### 2.6 Data Collection Tool and Procedure

A semi‐structured interviewer‐administered questionnaire was adopted from the literature by considering the local situation and the study's objective. The sampling procedure of the study participants is presented in Figure 1. Two BSc health officers served as supervisors, and eight health extension workers served as data collectors. Infertility (outcome variable) was diagnosed using four questions: (1) Would you want to have children? (2) Have you used any form of birth control in the past 12 months? (3) On average, how many times a week does your husband sleep with/have sexual intercourse? (4) Have you and your partner tried to achieve pregnancy for 12 months or longer? [10, 23, 24, 34]. The questionnaire consisted of 73 questions. Eight questions assessing factors related to socio‐demographics. The questionnaire consisted of 20 questions related to sexual and reproductive factors. Twenty‐six questions to assess social and behavioral factors 12 assessed medical and surgical factors, two anthropometric measurements, weight (Excellent mechanical weight scale) and height (Fazzini height measurement tool), were measured with standard measuring tools to calculate BMI as body weight (kg) per height (m^2^), and seven fertility‐related questions. Physical activity‐related factors were assessed using an International Physical Activity Questionnaire (IPAQ), which had a test‐retest reliability of = 0.91, and stress‐related factors were also assessed through the Perceived Stress Scale (PSS) questionnaire with reliability of (0.93) [31, 32] After the tools (IPAQ and PSS) were translated into local language (Amharic) and back‐translated to ensure accuracy, Cronbach's alpha was calculated to measure the internal consistency tools (alpha = 0.76 and 0.82, respectively, for IPAQ and PSS).

Data were collected from both (couples) separately. During the data collection period, homes that were found closed and/or either where partner was only present where visited one more time. Data were collected from women during the second visit for those couples who were not found together. Male‐related data were collected from females through phone calls. Finally, those houses closed during the second visit and those questionnaires not completed through phone calls were considered as non‐response.

### 2.7 Data Quality Assurance

To maintain the consistency of the data collection tool, the questionnaire was translated into the local language (Amharic) and then finally back to English. A pretest was conducted on 5% of the sample size, and training was provided to data collectors and supervisors. Experts checked the face validity of the tools. Cronbach's alpha was calculated to check the reliability of the tool (*α* = 0.68), which was acceptable. The supervisor and principal investigator supervise the correct implementation of the data collection procedure and check the completeness daily. Finally, the investigators carefully checked the collected data (the entire questionnaire) for completeness and consistency.

### 2.8 Data Processing and Analysis

After the data were collected, they were entered and coded using EPI Data version 4.6 software and exported to SPSS version 26 software for data cleaning and analysis. Descriptive statistics were used and the data were organized, processed, interpreted, and presented through text, tables, and figures.

Bi‐ and multivariable logistic regressions at 95% confidence intervals were used to determine the associations between the dependent and independent variables. After bi‐variable logistic regression model was done, variables with a *p*‐value < 0.25 at 95% CI were transformed into a multivariable logistic regression model, and a backward stepwise selection method was applied to obtain the final model. Hosmer–Lemeshow (0.477) and omnibus (0.000) tests were used to test goodness‐of‐fit. Multicollinearity was checked using collinearity diagnostic statistics through a variable inflation factor (VIF) < 10 (VIF = 6). Model fitness was evaluated using the Hosmer–Lemeshow goodness‐of‐fit test, and the model explained 34% of the variance in infertility (Nagelkerke pseudo *R*² statistic = 0.34). The odds ratio (OR) with 95% CI was calculated to measure the strength of the association between independent and outcome variables. All statistical tests were two‐sided, and those with a *p*‐value < 0.05 were considered statistically significant.

## 3 Results

### 3.1 Socio‐Demographic Characteristics

Eight hundred couples participated in this study, resulting in a response rate of 94.6%. The participants' mean age was 27.74 (SD = ± 6.49) for women and 33.7 (SD = ± 5.56) for men. Among the female respondents, 278 (34.75%) were in the age range of 18–24, while male respondents (304, or 38.0%) were found in the age group of 30–34 years. Rural residents made up approximately 682 (85.25%) of the respondents. About one‐third (29.9%) of female and 184 (23.0%) male respondents had an educational level of able to read and write, and college and above, respectively. Socio‐demographic result Table 1.

**TABLE 1:** Socio‐demographic characteristics of couples of reproductive age living in Merhabete Woredas, Northshewa, Ethiopia, 2023 (*N* = 800).

Variable	Category	Frequency (*N*)	Percent (%)
Female age	18–24 years old	278	34.75
	25–29 years old	81	10.12
	30–34 years old	144	18.01
	35–39 years old	179	22.37
	40–49 years old	118	14.75
Male age	18–24 years old	46	5.75
	25–29 years old	166	20.75
	30–34 years old	304	38.00
	35–39 years old	154	19.25
	40–49 years old	130	16.25
Residence	Urban	118	14.75
	Rural	682	85.25
Level of education (female)?	No formal education	216	27.00
	Read and write	239	29.90
	Primary school	203	25.40
	Secondary school	101	12.60
	College and above	41	5.10
Level of education (male)?	No formal education	159	19.87
	Read and write	167	20.88
	Primary school	162	20.25
	^^^Secondary school	128	16.00
	College and above	184	23.00
Occupational status (female)?	Government employee	69	8.60
	Housewife	383	47.90
	Daily laborer	105	13.10
	Merchant	121	15.10
	Farmer	122	15.30
Occupational status (male)?	Government employee	266	33.25
	Daily laborer	42	5.25
	Merchant	101	12.62
	Farmer	246	30.75
	No job	99	12.38
	Others	46	5.75

*Note:* Key: Other: priest, student.

### 3.2 Reproductive and Sexual Behavior

Out of the respondents, 416 (52.0%) had their first menstrual period between the ages of 13 and 15, with an average age of 14 (SD = ± 1.61), and 495 (61.9%) of females had married before the age of 20, with an average age of 19 (SD = ± 1.8). The mean marriage duration was 3.3 years (SD = ± 1.23), and 59.8% couples had 3–4 years of marriage duration in their current marriage or relationship. Four hundred forty (80.0%) and 692 (86.5%) female respondents had regular menses and adequate sexual intercourse frequency, respectively. The majority of female 659 (82.4%) and 210 (26.75%) male respondents had a history of contraception use. Over 376 (60.9%) of the female respondents had a parity of 1–2, with 221 (27.8%) and 245 (30.6%) couples having a history of abortion and stillbirth, respectively. One hundred fifty (18.75%) had a family history of infertility. Reproductive and sexual behavior results Table 2.

**TABLE 2:** Reproductive and sexual behavior characteristics among couples of reproductive age living in Merhabete Woredas, Northshewa, Ethiopia, 2023 (*N* = 800).

Variable	Category	Frequency (*N*)	Percent (%)
Age of menarche	<=12 years	184	23.00
	13–15 years	416	52.00
	>=16 years	200	25.00
Age of marriage	< 20 years	495	61.90
	>= 20 years	305	38.10
Marriage duration	1–2 years	181	22.60
	3–4 years	478	59.80
	>=5 years	141	17.60
Menstrual cycle	Irregular	160	20.00
	Regular	640	80.00
Menstrual blood flow	Minimal	669	83.60
	Moderate	90	11.30
	Heavy	41	5.10
Sexual frequency	Inadequate	108	13.50
	Adequate	692	86.50
Ever used contraceptive? (female)	Yes	659	82.40
	No	141	17.60
Type of contraception? (female)	Natural	61	9.30
	IUD	80	12.10
	Injectable	278	42.20
	OCP	126	19.10
	Condom	42	6.40
	Implant	72	10.90
Hormonal contraception use duration. (female)	< 6months	152	25.60
	6–12 months	175	29.50
	=> 12 months	266	44.90
Ever used any form of contraceptive? (male)	Yes	210	26.75
	No	590	73.25
Type of contraception (male)	Natural methods	79	37.80
	Condom	131	62.20
Parity	Nulli para	101	12.60
	1–2 parity	439	54.90
	>=3	260	32.50
Abortion history	Yes	221	27.60
	No	579	72.40
Stillbirth history	Yes	245	30.60
	No	555	69.40
Family history of infertility?	Yes	150	18.75
	No	650	81.25
If so, who was affected?	Mother	34	22.70
	Sister	47	31.30
	Both	21	14.00
	Grand mother	37	24.70
	Aunt	11	7.30

### 3.3 Social and Behavioral Characteristics of Participants

Out of 800 study participants, 635 (79.4%) of couples were substance users, of whom 50 (6.25%) female and 140 (17.5%) males were current smokers. There were 257 (39.0%), 107 (13.4%), and 103 (21.8%) female participants who drank five or more cups of coffee, ever chewed chat, and drank alcohol heavily, respectively. Additionally, 292 (47.2%) of the male participants were heavy drinkers. Seven hundred forty‐one (92.6%), 690(86.75%), and 101(12.6%) female respondents had vigorous intensity physical activity (VIPA), moderate‐intensity physical activity (MIPA), and high stress, respectively. Social and behavioral results Table 3.

**TABLE 3:** Social and behavioral characteristics of reproductive age couples living in Merhabete Woredas, Northshewa, Ethiopia, 2023 (*N* = 800).

Variables	Category	Frequency	Percent (%)
Substance use	Yes	635	79.40
	No	165	20.60
Smoking female	Current smokers	50	1.25
	Noncurrent smokers	750	98.75
Smoking male	Current smokers	140	17.50
	Noncurrent smokers	660	82.50
Ever take coffee female	Yes	662	82.75
	No	138	17.25
Coffee intake frequency	>=5 cups per day	257	39.00
	< 5 cups per day	405	61.00
Ever chewed chat	Yes	107	13.40
	No	693	86.60
Chat frequency	Monthly or less	63	58.90
	Weekly	38	35.50
	Daily or almost daily	6	5.60
Alcohol drink ever female	Yes	472	59.00
	No	328	41.00
Drinks per week female	Heavy	103	21.80
	Past year abstinence	369	78.20
Alcohol drink ever male	Yes	619	77.40
	No	181	22.60
Drinks per week male	Heavy	292	47.20
	Past year abstinence	327	52.80
VIPA	Yes	741	92.60
	No	59	7.40
MIPA	Yes	690	86.25
	Sedentary	110	13.75
Stress	Low	350	43.80
	Moderate	349	43.60
	High	101	12.60

*Note:* Key: VIPA, vigorous intensity physical activity; MIPA, moderate intensity physical activity.

### 3.4 Medical and Surgical Characteristics of Participants

Out of the total study participants, 101 (12.6%) couples (both female and male) had a history of STI, while 86 (22.3%) females and 51 (13.2%) males sought medical care for STI. Furthermore, 83 (10.4%) female and 70 (8.75%) male participants had a history of chronic diseases. Additionally, 62 (7.75%) and 43 (5.4%) female and male participants had surgical history on abdomen/laparotomy 34 (54.8%), and varicocele 25 (55.6%), respectively. A total of 597 (74.6%) female and 655 (81.9%) male participants had normal body index. Result on medical and surgical factors Table 4.

**TABLE 4:** Medical and surgical factors among couples of reproductive age living in Merhabete Woredas, Northshewa, Ethiopia, 2023 (*N* = 800).

Variables	Category	Frequency (*N*)	Percent (%)
Partners STIs history?	None	413	51.60
	Female	207	25.90
	Male	79	9.90
	Both	101	12.60
Treated for STIs?	No both	79	20.50
	Yes both	122	31.60
	Yes (female)	86	22.30
	No (female)	26	6.70
	Yes male	51	13.20
	No male	22	5.70
Chronic medical illness? (female)	Yes	83	10.40
	No	717	89.60
If yes which? (female)	DM	37	44.60
	Hypertension	24	28.90
	Epilepsy	16	19.30
	Psychiatric disorder	6	7.20
Chronic diseases history? (male)	Yes	70	8.75
	No	730	91.25
If yes which? (male)	DM	18	25.70
	Hypertension	30	42.90
	Epilepsy	12	17.10
	Psychiatric disorder	10	14.30
Surgical history? (female)	Yes	62	7.75
	No	738	92.25
Which surgery? (female)	Abdominal/laparotomy	34	54.80
	Pelvic	22	35.50
	Hysterectomy/oophorectomy	6	9.70
Surgery history (male)	Yes	43	5.40
	No	757	94.60
Which surgery? (male)	Pelvic	20	44.40
	Varicocele	25	55.60
Body weight for female	Normal	597	74.60
	Under	45	5.60
	Over	68	8.50
	Obese	90	11.30
Body weight for male	Normal	655	81.90
	Under	44	5.50
	Over	57	7.10
	Obese	44	5.50

### 3.5 Infertility Prevalence and Proportion of Treatment Seeking

The prevalence of infertility was 23.4% (95% CI: 20.4–26.5). From the total infertile couples 100 (53.5%) did not seek medical care and their main reason 57% for not seeking medical care, were thought that infertility was no treatment at all. Result on proportion of treatment seeking Table 5.

**TABLE 5:** Proportion of treatment seeking among couples of reproductive age living in Merhabete Woredas, Northshewa, Ethiopia, 2023 (*N* = 800).

Variable	Category	Frequency (*N*)	Percent (%)
Infertility treatment‐seeking	Yes	87	46.50
	No	100	53.50
Cause of infertility?	Female	29	33.33
	Male	20	23.00
	Both	15	17.24
	Unexplained	23	26.43
Why you didn't seek a medical help?	Thought no treatment	57	57.00
	No nearby health facility	33	33.00
	Other specify	10	10.00

*Note:* Key: Other: waiting for super natural power, thought as a sin, due to cultural and social barriers.

### 3.6 Factors Associated With Infertility

About 11 factors were taken into multivariable logistic regression, including age of female, education of female, education of male, abortion history, stillbirth history, STI history, heavy alcohol use, family history of infertility, menstrual irregularity, stress, and BMI of female. After adjustment for potential confounders, the age of the female partner, educational status of male, STI history, moderate and high stress, and overweight and obese BMI of the female were significantly associated with infertility at *p*‐value < 0.05.

The odds of infertility among couples with a female partner aged 30–34 years old were 3.1 times higher than females aged 18–24 years old (AOR = : 3.15; 95% CI = : 2.78–14.78; *p* < 0.02); aged 35–39 years also 6.4 times more likely to be infertile as compared to women of 18–24 years old (AOR = : 6.41 95% CI = : 2.78–14.78; *p* < 0.001). Similarly, couples with a female partner aged 40–49 years old were 7.3 times more likely to be infertile than couples aged 18–24 years old (AOR = : 7.30; 95% CI = : 2.97–17.92; *p* < 0.001). Male participants who had no formal education were 3.9 times more odds (AOR = :3.92; 95% CI = : 1.88–12.89; *p* < 0.002), to have infertility compared to male with educational level of college and above. Similarly, compared to males with an educational level of college or above, males with educational level of read and write had 2.7 times higher infertility risk (AOR = : 2.71; 95% CI = : 1.03–7.104; *p* < 0.02).

Female participants who had STI history had 3.2 times (AOR = : 3.20; 95% CI = : 2.23–8.09; *p* < 0.001) the odds of having infertility compared to those with no STI history. Compared to male participants with and without STI histories, those male partners with STI history had 1.91 times (AOR = : 1.91; 95% CI = : 1.80–9.73; *p* < 0.001) higher odds of having infertility. Couples with STI history had 3.8 times (AOR = : 3.82; 95% CI = : 2.18–9.78; *p* < 0.001) higher odds of infertility than couples without STI history. Couples with female spouses, who had moderate stress were 1.7 times (AOR = : 1.71; 95% CI = : 1.20–3.99; *p* < 0.02), more risk of having infertility when compared to their counterparts who had low stress. When compared to couples with low stress, those couples with female spouses who had high stress had 3.9 times (AOR = : 3.97; 95% CI = : 3.44–15.38; *p* < 0.001) greater probability of infertility.

Compared to female participants with a normal BMI and an over‐weight BMI, the over‐weighted spouses had 2.7 times (AOR = : 2.73; 95% CI = : 1.145–6.521; *p* < 0.01) more odds of infertility than those with a normal BMI. Furthermore, obese women had 3.1 times (AOR = : 3.12; 95% CI = : 1.77–8.21; *p* < 0.00) higher likelihood of developing infertility compared to women with a normal BMI. Associated factors with infertility Table 6.

**TABLE 6:** Bi‐variable and multivariable analysis of factors associated with infertility among couples of reproductive age living in Merhabete, Northshewa, Ethiopia, 2023 (*N* = 800).

Variables	Category	Frequency (%)		COR (95% CI)	AOR (95% CI)	*p*‐value
		Fertile	Infertile			
Female age	18–24 years	259 (93.2)	19 (6.8)	1	1	
	25–29 years	62 (76.5)	19 (23.5)	4.17 (2.08–8.35)	1.64 (0.55–4.88)	0.15
	30–34 years	116 (80.6)	28 (19.4)	3.29 (1.76–6.13)	3.15 (1.24–7.98)	**0.02**
	35–39 years	111 (62.0)	68 (38.0)	8.35 (4.79–14.78)	6.41 (2.78–14.78)	**0.001**
	40–49 years	65 (55.1)	53 (44.9)	11.11 (6.15–20.06)	7.3 (2.97–17.92)	**0.0002**
Female education	No formal education	136 (63.0)	80 [35]	2.42 (1.06–5.51)	0.85 (0.25–2.89)	0.25
	Read and write	187 (78.2)	52 (21.8)	1.14 (0.50–263)	1.15 (0.32–4.11)	0.10
	Primary school	175 (86.2)	28 (13.8)	0.66 (0.27–1.57)	0.43 (0.12–1.59)	0.13
	Secondary school	82 (81.2)	19 (18.8)	0.95 (0.38–2.39)	0.71 (0.18–2.70)	0.17
	College and above	33 (80.5)	8 (19.5)	1	1	
Male education	No formal education	101 (63.5)	58 (36.5)	4.70 (2.67–8.28)	3.92 (1.88–12.89)	**0.002**
	Read and write	119 (71.3)	48 (28.7)	3.30 (1.86–5.86)	2.71 (1.03–7.10)	**0.02**
	Primary school	120 (74.1)	42 (25.9)	2.87 (1.60–5.13)	1.13 (0.38–3.38)	0.11
	Secondary school	109 (85.2)	19 (14.8)	1.42 (0.72–2.80)	1.31 (0.44–0.61	0.14
	College and above	164 (89.1)	20 (10.9)	1	1	
Menstrual cycle	Irregular	113 (70.6)	47 (29.4)	1.48 (1.00–2.19)	1.47 (0.97–3.59)	0.07
	Regular	500 (78.1)	140 (21.9)	1		
Stillbirth	Yes	144 (69.2)	64 (30.8)	2.95 (1.96–4.45)	1.70 (0.95–3.017)	0.23
	No	359 (86.9)	54 (13.1)	1	1	
Family history	Yes	104 (69.3)	46 (30.7)	1.59 (1.07–2.36)	1.37 (0.72–2.60)	0.11
	No	509 (78.3)	141 (21.7)	1	1	
STIs history	None	509 (78.3)	141 (21.7)	1	1	
	Female	136 (65.7)	71 (34.3)	3.26 (2.18–4.86)	3.20 (2.23–8.09)	**0.000**
	Male	59 (74.7)	20 (25.3)	2.11 (1.18–3.77)	1.91 (1.80–9.73)	**0.001**
	Both	62 (61.4)	39 (38.6)	3.92 (2.41–6.40)	3.82 (2.18–9.78)	**0.001**
Stress	Low stress	297 (84.9)	53 (15.1)	1	1	
	Moderate stress	267 (76.5)	82 (23.5)	1.72 (1.17–2.52)	1.71 (1.20–3.99)	**0.02**
	High stress	49 (48.5)	52 (51.5)	5.94 (3.65–9.68)	3.97 (3.44–15.38)	**0.001**
BMI of female	Normal weight	498 (83.4)	99 (13.6)	1	1	
	Under weight	27 (60.0)	18 (40.0)	3.35 (1.77–6.32)	1.25 (0.47–3.31)	0.10
	Over weight	34 (50.0)	34 (50.0)	5.03 (2.98–8.47)	2.73 (1.14–6.52)	**0.012**
	Obese	54 (60.0)	36 (40.0)	3.35 (2.08–5.38)	3.12 (1.77–8.21)	**0.000**

*Note:* Bold *p*‐values are significantly associated factors.

## 4 Discussions

In this study, the overall prevalence of infertility was 23.4% (95% CI: 20.4–26.5). This finding was in line with the study done in Nigeria 26.5% [36]. This might be due to both studies were performed in countries thought to have similar health system plans and have comparable socioeconomic status.

The prevalence of infertility in this study was higher than the 12‐month international estimate for developing countries (9.2%–9.3%) and the global prevalence of 10%–15% [4, 37], as well as compared to 15.5% in the United States [35], and 15.7% in Canada [38]. This may be due to higher rates of STIs, abortion history, and self‐reported infertility without clinical confirmation in our study area. Our findings were also higher than reports from Iran (4.73%) [34], China 15.5% [10], Saudi Arabia 18.93% [39], and Gambia 14.3% [40]. Possibly due to differences in study populations, age ranges, and community‐based vs. clinic‐based settings. Cameroon, Douala 19.2% [41], and Ouagadougou (Burkina Faso) (9.3%) [42]. The higher prevalence in this study could reflect a larger sample size, methodological differences, and socio‐demographic factors.

In contrast, the prevalence of infertility in this study was lower than a study conducted in developing countries, which showed that overall infertility in Africa was 30% [11]. Differences in infertility definitions and study settings may explain variations, such as the lower prevalence compared to Addis Ababa (27.6%) with a smaller, institution‐based sample [23].

This study found that higher female age was strongly associated with infertility. Women aged 30–34, 35–39, and 40–49 years had 3, 6, and 7 times higher risk of infertility, respectively, compared to those aged 18–24 years, with findings from the United States [35], Canada [38], Turkey [43], and Morocco [44] support this finding. This may be due to age‐related declines in both the quantity and quality of a woman's ovarian reserve, with monthly pregnancy chances dropping from 20% at age 30 to 5% at age 40 [41]. In addition, as age increases the risk of getting STIs, stress, other chronic medical problems, and exposure to harmful environmental pollutants also rises [4, 41]. This all might be associated with infertility among couples.

Those male partners who had no formal education, and who were able to read and write had 3.9 times and 2.7 times more likely to have infertility, respectively, as compared to their counterparts who had educational levels of college and above. This result was argued with studies done in China [10], Turkey [43], Iraq [34], and Morocco [44]. Higher‐educated partners may have greater awareness of healthy lifestyles, STI prevention, and early treatment, and are more likely to seek information about infertility and its risk factors. In contrast, males with higher educational levels were associated with higher infertility from a study performed in Canada [38]. These contradictory findings may reflect urbanization, delayed marriage, and lifestyle factors among educated partners, which can reduce childbearing and increase infertility risk. Further research is needed to clarify these relationships [45].

Participants with a history of STIs (female, male, or both partners) had 3.2 times, 1.9 times, and 3.8 times greater likelihood of infertility, respectively, as compared to their contradictory participants, who had no STI history. Research from Scotland [46], Egypt [47], Cameroon [41], and Dessie, Ethiopia [48], supports this finding. STIs, reduced sexual desire, pain, and vaginal discharge can lead to pelvic inflammatory disease if untreated, causing damage and scarring of the reproductive tract, fallopian tubes, uterus, and surrounding tissues, as well as reduced sperm motility, ultimately related with the risk of infertility, particularly tubal infertility [4, 45].

Moderate and high stress were significantly associated with infertility, with odds 1.7 and 3.9 times higher, respectively, compared to low‐stress couples, consistent with findings from low‐ and middle‐income countries [20], and India [49]. Stress can disrupt the hypothalamic‐pituitary‐ovarian axis, altering reproductive hormones and menstrual cycles, and related with ovulatory dysfunction and infertility. Mind‐body interventions, such as psychological and social support and exercise, are recommended to reduce stress in the community [50].

Infertility risk was higher among couples with overweight or obese female partners, being 2.7 and 3.1 times greater, respectively, compared to those with normal BMI. This result was similar to the study findings reported from China [51], Iran [52], Turkey [53], Scotland [46], Italy [54], and India [55]. Obesity in women can disrupt metabolism and ovarian function, and related with ovulatory disorders and reduced fertility. Higher BMI is also associated with lower embryonic implantation rates, with pregnancy per cycle at 38.3% in obese women vs. 45.5% in non‐obese women [56]. Most female partners in this study were housewives with low education, likely associated with less physical activity. Regular exercise and maintaining a healthy weight can help reduce infertility.

Even though infertility prevalence in this study was high, less than half of affected couples (46.5%, 95% CI: 37–52.9) sought treatment. This is similar to findings from China (≈50%) [10] and France (42%) [57], but lower than in Britain (57.3%) [58]. The low treatment‐seeking behavior may be influenced by multiple factors, including limited health literacy, socioeconomic constraints, and restricted availability of infertility services—especially assisted reproductive technologies—which are concentrated in a few urban centers. In addition, cultural norms, social stigma, and fear of marital or community judgment may discourage couples from openly seeking care. Collectively, these structural, economic, and socio‐cultural barriers likely contribute to the observed low utilization of infertility services [41].

### 4.1 Strengths and Limitations of the Study

As strength, this study included both partners and conducted at the community level to assess the proportion of infertility treatment seekers in addition to prevalence and associated factors however; it has also several limitations. First, the cross‐sectional design precludes causal inference, and temporal relationships between exposure variables and infertility cannot be established. Second, data were self‐reported, which may introduce recall bias and social desirability bias, particularly for sensitive variables such as sexual behavior and reproductive history, and chronic illness and medical conditions. Third, some of the associations identified in this study have wide confidence intervals, likely due to small sample sizes in certain subgroups. This may reduce the precision of the estimated effects. Finally, this study didn't differentiate between primary and secondary infertility, but these limitations should be considered when interpreting the findings.

## 5 Conclusions and Recommendations

The prevalence of infertility in this study was high compared to the WHO worldwide estimate, with about half of them not seeking medical care for this problem. This high prevalence of infertility was associated with female age, male education, STI, stress, and BMI of the female partner. Unless emphasis is given to infertility, it will result in more public health problems. Therefore, based on the identified associated factors, the following evidence‐based and context‐appropriate interventions are recommended: active health education, and the provision of diagnostic and treatment services for this community, Integration of Infertility Screening into Primary Healthcare Services. Given the significant association between STI history and infertility, strengthening STI prevention strategies, including community awareness, routine screening, partner notification, and early treatment—should be prioritized within reproductive health programs. The minister of health should design and align infertility interventions with existing reproductive health platforms and health education programs aimed at infertility, its associated factors, and benefits of infertility treatment seeking to reverse fertility. Future researchers will assess the prevalence of infertility and its associated factors using clinical diagnostic modalities.

## Author Contributions

**Hailegiyorgis Geleta Abocherugn:** investigation, methodology, project administration, data curation, software, writing – original draft, writing – review and editing, resources, conceptualization. **Abera Mamo Dibabu:** conceptualization, investigation, writing – original draft, methodology, validation, visualization, supervision. **Desalegn Girma:** project administration, resources, conceptualization, formal analysis, writing – review and editing, investigation, writing – original draft. **Amare Workie Gashu:** conceptualization, validation, software, project administration. **Melese Wagaye Zergaw:** conceptualization, investigation, validation, methodology, software, data curation.

## Funding

The authors have nothing to report.

## Disclosure

The lead author Hailegiyorgis Geleta Abocherugn affirms that this manuscript is an honest, accurate, and transparent account of the study being reported; that no important aspects of the study have been omitted; and that any discrepancies from the study as planned (and, if relevant, registered) have been explained.

## Ethics Statement

All methods were carried out in accordance with relevant guidelines and regulations and ethical approval and clearance were obtained from the Institutional Research Ethics Committee (IRC) of Mizan‐Tepi University, College of Medicine and Health Sciences with an approval code of: HSC/00505/2014 E.C on the date of 13.07/2014 E.C. A legal supportive letter was written from the midwifery department to Merhabte Woreda Health Office. Individual informed written consent was obtained from each respondent after explaining the purpose of the study and his or her participation was voluntary with the right to withdraw from the study any time without any penalty. All the information obtained from the participants was kept confidentially by using codes rather than their names and using these data only for this research.

## Conflicts of Interest

The authors declare no conflicts of interest.

## Data Availability

The data that support the findings of this study are available from the corresponding author upon reasonable request.
